# Effects of transcranial direct current stimulation combined with listening to preferred music on memory in older adults

**DOI:** 10.1038/s41598-021-91977-8

**Published:** 2021-06-16

**Authors:** Ricky Chow, Alix Noly-Gandon, Aline Moussard, Jennifer D. Ryan, Claude Alain

**Affiliations:** 1 Rotman Research Institute, Baycrest Centre, Toronto, ON Canada; 2 Department of Psychology, University of Toronto, Toronto, ON Canada; 3 Department of Psychiatry, University of Toronto, Toronto, ON Canada; 4 Institute of Medical Science, University of Toronto, Toronto, ON Canada; 5 Music and Health Science Research Collaboratory, University of Toronto, Toronto, ON Canada

**Keywords:** Cognitive ageing, Learning and memory

## Abstract

Listening to autobiographically-salient music (i.e., music evoking personal memories from the past), and transcranial direct current stimulation (tDCS) have each been suggested to temporarily improve older adults’ subsequent performance on memory tasks. Limited research has investigated the effects of combining both tDCS and music listening together on cognition. The present study examined whether anodal tDCS stimulation over the left dorsolateral prefrontal cortex (2 mA, 20 min) with concurrent listening to autobiographically-salient music amplified subsequent changes in working memory and recognition memory in older adults than either tDCS or music listening alone. In a randomized sham-controlled crossover study, 14 healthy older adults (64–81 years) participated in three neurostimulation conditions: tDCS with music listening (tDCS + Music), tDCS in silence (tDCS-only), or sham-tDCS with music listening (Sham + Music), each separated by at least a week. Working memory was assessed pre- and post-stimulation using a digit span task, and recognition memory was assessed post-stimulation using an auditory word recognition task (WRT) during which electroencephalography (EEG) was recorded. Performance on the backwards digit span showed improvement in tDCS + Music, but not in tDCS-only or Sham + Music conditions. Although no differences in behavioural performance were observed in the auditory WRT, changes in neural correlates underlying recognition memory were observed following tDCS + Music compared to Sham + Music. Findings suggest listening to autobiographically-salient music may amplify the effects of tDCS for working memory, and highlight the potential utility of neurostimulation combined with personalized music to improve cognitive performance in the aging population.

## Introduction

Research into the arousal-and-mood hypothesis has suggested that listening to enjoyable music can heighten arousal and induce positive affect, thereby influencing subsequent cognitive performance^1,2^. In older adults, benefits following music listening were shown in various cognitive domains such as autobiographical memory recall^3^, working memory^4^, semantic fluency^5^, semantic memory^6^, and episodic memory^7–9^. Given individual differences in musical listening preferences and distastes, autobiographically-salient music may be of particular interest in subsequently eliciting cognitive benefits following music listening. Music that evokes autobiographical memories or holds substantial personal significance for an individual (i.e., autobiographically-salient music) can be especially powerful in evoking strong positive affect and heightened arousal^10,11^. For older adults, listening to autobiographically-salient music heightens positive affect and evokes feelings of nostalgia and idiosyncratic meaningful memories, arising from emotionally salient associations formed between particular songs and events from their younger years^11–14^. As healthy aging is associated with declines in cognitive domains such as working memory and episodic memory^15,16^, listening to autobiographically-salient music, according to the arousal-and-mood hypothesis, may have potential to improve short-term performance in these cognitive domains by improving positive affect and heightening one’s arousal and emotional state.

Similarly, non-invasive brain stimulation has shown potential in temporarily improving working memory and episodic memory^17^. One such technique is transcranial direct current stimulation (tDCS), which involves applying a low-intensity current flow via a positive electrode (anode) and a negative electrode (cathode) placed atop the scalp. Current from anodal tDCS is thought to temporarily heighten cortical excitability at the targeted stimulation site by modulating the resting membrane potential of neurons^18,19^. The resulting increase in cortical excitability may lead to short- and long-term behavioural changes observed post-stimulation and is thought to be attributed to long-term potentiation and cortical plasticity^20^. The left dorsolateral prefrontal cortex (DLPFC) is a common stimulation site for investigating improvement in performance for working memory and episodic memory tasks. In healthy older adults, several studies using tDCS have demonstrated subsequent improvements in working memory^21,22^ and episodic memory^23–26^ after administration of tDCS to the DLPFC, even after a single stimulation session^23,27,28^. For example, older adults’ accuracy in a verbal n-back task improved after a single 30-min stimulation session of 2 mA anodal tDCS over the left DLPFC compared to sham stimulation^28^. However, the consensus remains mixed as to the reliability of tDCS by itself to improve cognition in healthy older adults, with some studies finding no effect of prefrontal tDCS on cognitive abilities^29–31^. Furthermore, these mixed findings are also interleaved with studies using different parameters, tasks, and outcome measures during or following stimulation, as well as considering the high variability in individuals’ responsivity to tDCS^32^. A meta-analysis^17^ demonstrated cognitive benefits following tDCS in healthy older adults with a moderate effect size (0.42), while another meta-analysis concluded no evidence of cognitive benefits in healthy adults from 18–50 years of age following tDCS^33^. Although tDCS has shown promise for alleviating cognitive impairments in older adult clinical populations such as those with dementia^17^, it is less clear what conditions enable tDCS to reliably demonstrate maximal responsivity in the healthy aging population^30,32^.

As prior research has suggested tDCS and autobiographically-salient music may independently confer benefits in subsequent memory in older adults, one may expect gains in memory to be amplified when combined together. One study^34^ investigated the effects of anodal tDCS on the DLPFC on inhibitory control in younger adults using a Stop-Signal task while concurrently listening to either silence, low-tempo, or high-tempo background music, finding that high-tempo background music interacted with the effects of tDCS on response inhibition. Another study^35^ investigated the effects of cerebellar tDCS followed by music listening on a Line Bisection Task in younger adults, revealing that the pairing of cathodal (inhibitory) stimulation and music listening compensated for pseudoneglect in visuospatial attention. No study to our knowledge has examined changes in memory performance following anodal tDCS and listening to autobiographically-salient music in older adults. The aim of the present study was to investigate whether anodal tDCS with concurrent listening to autobiographically-salient music amplified older adults’ subsequent memory performance than either tDCS in silence or music listening under sham stimulation in two domains of memory: working memory and recognition memory.

Anodal tDCS was applied over the left DLPFC using a current intensity of 2 mA and stimulation length of 20 min based on prior literature demonstrating improvements in working memory and episodic memory following these stimulation parameters^24,28,34^. Working memory was measured pre- and post-stimulation using a digit span task, and episodic memory was measured post-stimulation using an auditory word recognition task (WRT) during which neuroelectric activity was recorded by electroencephalography (EEG). We expected changes in working memory and recognition memory to be amplified when tDCS was concurrently administered to participants listening to autobiographically-salient music relative to either sham stimulation with music listening or tDCS administered in silence.

## Methods

### Participants

Participants were recruited if they were between 60 to 85 years of age, reported normal or corrected-to-normal vision and hearing, no history of learning disabilities, stroke, transient ischemic attack, traumatic brain injury with loss of consciousness greater than 5 min, substance abuse disorder, neurodegenerative disease, brain abnormalities, history of intracranial surgery, seizure disorder, or any other diagnosis of major neurological or psychiatric disorder. Exclusion criteria also included concurrent treatment with ototoxic medicines, medications affecting cognitive abilities, presence of implanted devices, or traces of metal in the body. Seventeen older adults participated in this study. Data were excluded from analysis from two participants due to attrition, and from another participant because of insufficient length of stimulation time in one condition owing to technical difficulties during tDCS. The final sample comprised 14 older adults (64–81 years, 11 females, see Table 1). All participants were right-handed and had no prior experience with tDCS or any of the experimental tasks. Individuals were recruited from the participant database at the Rotman Research Institute as well as through advertisements. The study protocol was approved by the Research Ethics Board of the Rotman Research Institute at Baycrest Centre. All participants were informed about the procedures and possible risks of tDCS, and informed written consent was obtained from all participants. All methods were performed in accordance with the relevant guidelines and regulations, including safety protocols based on established guidelines on tDCS administration^36^. Participants received monetary compensation for their participation in the study.

**Table 1:** Demographic, clinical, and hearing assessment data.

Variable	Mean (SD) (*n* = 14)
Age (years)	72.64 (4.89)
Education (years)	16.29 (3.38)
Gender (F:M)	11:3
Average bilateral PTA threshold (Hz)	16.07 (5.51)
QuickSIN (SNR loss)	2.09 (1.31)
WAIS-III Digit Span Total (scaled score)	12.33 (3.18)

Digit span scaled scores displayed were acquired from participants’ first visit pre-stimulation.

*PTA* pure tone audiogram, *SNR* signal-to-noise ratio, *WAIS-III* Wechsler Adult Intelligence Scale, Third Edition.

### Pre-experiment musical listening interview

Prior to study participation, participants were asked about musical listening preferences and past musical experiences to ensure autobiographical saliency of the music to be played during the study. During the interview, participants were probed for their preferred genres of music and to share their favorite songs and artists from their past and present. Participants were also probed for music listening preferences throughout each decade of their lifespan starting from childhood and teenage years, as well as specific songs (both song names and their respective artists) that held considerable idiosyncratic meaning or evoked personal memories of the past (e.g., wedding songs, or songs that reminded them of a trip they made, or those they shared with close friends). This information was then used for experimenters to compile a personalized playlist of songs for each participant. The playlist was procured such that its entire length from start to finish lasted approximately the same time window of the stimulation phase (21 min). Specific songs that were probed through the interview were prioritized for inclusion in each playlist, and the remaining time allotted to the playlist included songs that represented their favorite musical artists and genres from their past. Each playlist thus contained both participant-selected music and music selected by experimenters based on participants’ individual preferences. Playlists were curated on Spotify, and recorded versions of songs were selected over live versions. Within and across playlists, the included songs were variable in time period and musical genre, spanning classical, rock, jazz, folk, pop, country, and film score music. Playlists included both vocal and instrumental-only songs, although most songs within the majority of playlists were vocal in nature.

### Experimental design and procedure

A counterbalanced and crossover (repeated-measures) design was used where all participants underwent three testing sessions, each with a different neurostimulation condition, with each session conducted at least one week apart to prevent carry-over effects^37^. In the *tDCS-only* condition, participants received anodal tDCS in silence. In the *tDCS* + *Music* condition, participants received anodal tDCS while simultaneously listening to autobiographically-salient music. In the *Sham* + *Music* condition, participants received sham tDCS stimulation while simultaneously listening to autobiographically-salient music. The order of the three conditions was randomized and counterbalanced across participants, and participants were blind to Sham + Music and tDCS + Music conditions. To minimize circadian influences on performance, all three testing sessions for each participant were scheduled at the same time of day^38,39^.

During the first study visit, all participants completed demographic questionnaires pertaining to health and musical experiences, a brief hearing assessment comprising pure tone audiograms for octave frequencies from 250 to 8000 Hz, and the QuickSIN test (Etymotic Research Inc, Elk Grove, IL, USA) to ensure normal hearing thresholds and speech-in-noise listening abilities for their age. Participants then completed measures of self-reported emotional affect and working memory. The stimulation phase followed, involving one of tDCS-only, tDCS + Music, or Sham + Music conditions. In the conditions involving music listening, each song from the personalized playlist was played in its entirety. The order of songs was randomized for each participant’s playlist between the tDCS + Music and Sham + Music sessions. Autobiographically-salient music was played through soundbooth speakers at an individual comfortable listening level that was kept constant for each participant’s two music listening conditions. Immediately post-tDCS, participants completed another measure of emotional affect and working memory. Following these measures, participants completed the auditory WRT that measured verbal recognition memory while neuroelectric activity was recorded using EEG. A 15-min time window was allocated between the end of the stimulation phase and the start of the auditory WRT to ensure sufficient time for EEG setup. Auditory brainstem responses to speech phonemes were recorded following the WRT, and those data will be reported elsewhere. Participants completed the remaining stimulation conditions on separate visits with the same procedure but without the initial hearing assessments from the first study visit. All testing sessions were conducted while the participant was seated comfortably in a recliner inside an electromagnetically-shielded double-walled sound-attenuated booth.

### Measure of emotional affect

The Positive Affect and Negative Affect Schedule^40^ (PANAS), a self-report questionnaire, was used to measure changes in emotional affect pre- and post-tDCS. Participants were to score a list of ten adjectives of positive affect (e.g., “excited,” “enthusiastic”) and ten adjectives of negative affect (e.g., “ashamed,” “hostile”) on a five-point Likert scale (from 1 denoting “not at all” to 5 denoting “extremely”) according to the extent to which the participant was experiencing the given emotion at that particular moment in time.

### Measure of working memory

The digit span forward and backward subtests from the Wechsler Memory Scale, Third Edition (WMS-III)^41^ were used to measure changes in working memory pre- and post-tDCS. In the digit span forward subtest, participants heard a string of digits spoken by an experimenter at a rate of 1 digit per second, and were instructed to repeat back the string verbatim. In the backward subtest, participants similarly heard a string of digits spoken by an experimenter, but were to repeat the string in the backwards order. For both subtests, strings of digits gradually increased in span until the participant incorrectly repeated two strings of the same length. The digit span tasks were administered and scored in accordance with instructions from the WMS-III administration manual: a score of 1 was given if participants could report each string of digits verbatim, and a score of 0 was given if they could not. The digit span was always administered immediately following completion of the PANAS in all stimulation conditions, and the same digits were read in each condition.

### Transcranial direct current stimulation

A constant direct current (2 mA, 20 min) was administered by a battery-driven constant current stimulator from TCT Research (TCT Research Limited, Kowloon, Hong Kong) through 35 cm^2^ saline-soaked synthetic sponge electrodes. The anode was placed over the left DLPFC, located over the F3 electrode location according to the International 10–20 system of EEG electrode placement. Evidence for using F3 for targeting the left DLPFC is supported by models of current flow intensity and distribution^42^. The cathode was placed over the contralateral supraorbital region. The stimulation phase took 21 min. At the start of the stimulation phase, the current gradually increased in a ramp-like fashion from 0 to 2 mA over 60 s, remained constant for 19 min and 50 s, then was ramped down to 0 mA over 10 s. In the Sham + Music condition, current also gradually increased to 2 mA over a time window of 60 s, then immediately ramped down to 0 mA over a 10 s period where it was maintained at that level of current for 20 min until the end of the stimulation phase. To ensure that no participant experienced adverse side effects, a post-stimulation questionnaire was administered after each stimulation phase composing of Likert scales for a variety of side effects from “0” being absent to “4” being severe.

### Measure of recognition memory

The auditory WRT was used to assess verbal recognition memory following each stimulation phase. Participants heard a continuous stream of everyday spoken words (e.g., “glasses,” “forests,” “whisper”), and were instructed to assess whether each word was old (i.e., previously heard within the context of the task on the day of their visit) or new (i.e., never heard within the context of the task on the day of their visit). Participants responded by pressing the numeric “1” key on a computer keypad with their right index finger for words judged as new, and the numeric “2” key with the right middle finger for words judged as old. Participants were instructed to respond as quickly and accurately as possible.

Three hundred word stimuli were recorded by two male and two female native speakers of North American English. The stimuli were randomly separated into three word lists of 100 words each using MATLAB software, one list for each of the three experimental blocks of trials. No stimuli were shared between the three word lists. Thirty words out of each of the three 100-word lists were randomly assigned to the “old” condition and distributed into three repetition lags, with a single repetition after 1, 3, or 7 intervening words after its initial presentation (R2, R4, or R8 repetition lags respectively). Thus the entire stimulus set comprised 210 unique word stimuli: 30 words for each of the three repetition lags (90 unique words presented twice) and 120 unique words presented only once. The order of the three experimental blocks was pseudo-counterbalanced for each participant’s visit (i.e., list order for the first visit was 1, 2, 3; second visit, 2, 1, 3; third visit, 3, 1, 2). Stimuli were binaurally presented through Etymotic ER-3A insert earphones (Etymotic Research, Elk Grove, IL, USA) at 76 A-weighted decibels sound pressure level. The WRT consisted 100 trials for each of the three experimental blocks and lasted approximately 15 min. Each trial was presented with a stimulus-onset asynchrony of 3000 ms. The experiment was administered using Presentation software (Version 16.3, Neurobehavioral Systems, Inc, Berkeley, CA). Participants did not receive any feedback on their performance during the task.

### EEG acquisition and preprocessing

Neuroelectric activity was recorded from Fpz, Fz, Cz, Pz, P3, P4, TP9, and TP10 electrodes at a 512 Hz sampling rate using a BioSemi ActiveTwo acquisition system (BioSemi V.O.F., Amsterdam, Netherlands). The electrode montage was based on the standard 10–20 system, and used a pair of common mode sense and driven right leg reference electrodes. The EEG was re-referenced to linked mastoids (i.e., the average of TP9 and TP10 electrodes) for analysis of event-related potentials (ERPs). EEG preprocessing was performed by experimenters who were blind to condition. For each participant, principal component analyses from EEGLab^43^ were used to identify a set of ocular movements from the continuous EEG and then used to generate spatial components that best accounted for artifacts from eye movements. The spatial topographies were then subtracted from the continuous EEG to correct for data contaminated from eye-blinks. The continuous EEG was digitally band-pass filtered to attenuate frequencies below 0.53 Hz (forward, 6 dB/octave) and above 30 Hz (zero phase, 24 dB/octave). Using Brain Electrical Source Analysis software (BESA Research 7.1, MEGIS GmbH, Gräfelfing, Germany), the EEG data were visually inspected to identify segments contaminated by defective electrodes if present. Data for each participant were then segmented into epochs of 0–1500 ms in length with a baseline of 500 ms prior to stimulus onset. Only the epochs for which participants responded correctly (hits and correct rejections) were included in the analyses. Epochs were scanned for additional artifacts, with those including deflections exceeding 50 μV marked and excluded from the analysis. This excluded an average of 3.89% of trials per participant in tDCS-only, 2.71% in Sham + Music, and 2.95% in tDCS + Music, which did not significantly differ between conditions, *F*(2, 26) = 0.50, *p* = 0.611, *η*_*p*_^2^ = 0.037. The remaining epochs were averaged according to experimental conditions, and averaged epochs were baseline-corrected with respect to the pre-stimulus interval (i.e., mean amplitude over the 500 ms prior to stimulus onset).

### Data analysis

Neural and behavioural measures were subjected to analyses of variance (ANOVA) with Sidak correction to compensate for multiple comparisons. Partial eta-squared was calculated as a measure of effect size. A Greenhouse–Geisser correction was used when the assumption of sphericity had been violated. An alpha value of 0.05 was used throughout, and statistical analyses were conducted using SPSS (IBM SPSS Statistics 26.0; Ehningen, Germany).

#### Behavioural measures

PANAS ratings were summed across positive and negative affect scales. Preliminary analyses for summed ratings revealed floor effects for the negative affect scale, with the majority of summed ratings (81%) at a value of 10 (lowest summed rating for negative affect) and exhibited little variability, and so this scale was excluded from analysis. Ratings for the positive affect scale were subjected to a two-way repeated-measures ANOVA with within-subject factors of Condition (tDCS-only, tDCS + Music, Sham + Music) and Time (pre-stimulation, post-stimulation). Forward and backward digit span scores were subjected to an ANOVA with the same within-subject factors. For the auditory WRT, trials with a null response or that exceeded 3000 ms from stimulus onset were excluded from the analysis. Accuracy was calculated from the hit rate minus false alarm rate for old words, and RT values were calculated only from hits and correct rejections. Accuracy values were subjected to a repeated-measures ANOVA with within-subject factors of Condition (tDCS-only, tDCS + Music, Sham + Music) and Repetition Lag (R2, R4, R8), and RT values were subjected to a repeated-measures ANOVA with within-subject factors of Condition (tDCS-only, tDCS + Music, Sham + Music) and Trial Type (New, R2, R4, R8). Ratings for the tDCS side effects questionnaire following stimulation were subjected to a one-way repeated-measures Bayesian ANOVA using JASP software (Version 0.14.1)^44^ with default prior probabilities and with Condition (tDCS-only, tDCS + Music, Sham + Music) as a within-subjects factor. We denote a Bayes factor (B_10_) greater than 3 as indicating support for the alternative hypothesis (i.e., 3:1 odds in favor of the alternative)^45^. Conversely, a B_10_ less than 0.33 indicates support for the null hypothesis (3:1 odds in favor of the null).

#### ERPs

Figure 4 displays ERP waveforms of the auditory WRT by trial type. All words generated N1 and P2 deflections at central and frontal sites that peaked at about 125 and 250 ms respectively after word onset. In addition, an “old-new” effect modulation was evident in the late positive complex (LPC) over centro-parietal electrodes. The LPC, or “P600”, has been reported in previous studies of visual and auditory recognition memory tasks^46–48^, has a central posterior scalp distribution, and is thought to index episodic memory recollection^49–52^. Peak latency for the LPC was defined as the maximum positive voltage over a time window of 350–900 ms. Peak latencies and mean amplitudes for the LPC were derived from, and averaged over, a pre-defined cluster of centro-parietal electrodes (Cz, Pz, P3, P4). Mean amplitudes for each trial type were averaged over different time windows based on visual inspection of average waveforms for each repetition lag. LPC mean amplitudes were averaged over a 400 ms time window centred on the grand peak latency per condition: 600–1000 ms for correctly recognized new words, 450–850 ms for correctly recognized old words from R2, 500–900 ms from R4, and 550–950 ms from R8. Peak latencies and mean amplitudes from the LPC were subjected to a repeated-measures ANOVA with within-subject factors of Condition (tDCS-only, tDCS + Music, Sham + Music) and Trial Type (New, R2, R4, R8). As the primary measure of interest for mean amplitudes was the old-new effect (i.e., the amplitude difference between old and new trial types), separate post-hoc univariate ANOVAs examined the old-new amplitude difference between Condition for each repetition lag.

## Results

### Demographic and clinical data

Participant demographics and hearing assessment data are displayed in Table 1. In response to questions about their music listening habits, all participants reported that memories of past events of their lives occasionally come to mind when listening to specific songs or musical genres in their everyday life. All participants also reported experiencing some sort of emotion (e.g., sadness, happiness, nostalgia, enthusiasm) when listening to music in general. Of the sample, three participants identified as musicians, and the 11 other participants identified as non-musicians. Participants demonstrated normal or near-normal levels of hearing acuity and speech-in-noise comprehension expected for older adults. Scaled scores of the total digit span collected from participants’ first study visit pre-stimulation (*M* = 12.33*, SD* = 3.18) indicated that baseline level of working memory performance from our older adult sample was comparable to norms expected for their age. Very brief mild side effects such as tingling, mood changes, and neck stiffness were reported during and following anodal and sham tDCS. The Bayesian analysis for ratings of side effects experienced post-stimulation demonstrated support for a null difference in ratings between conditions, B_10_ = 0.253.

### PANAS

**Figure 1:**
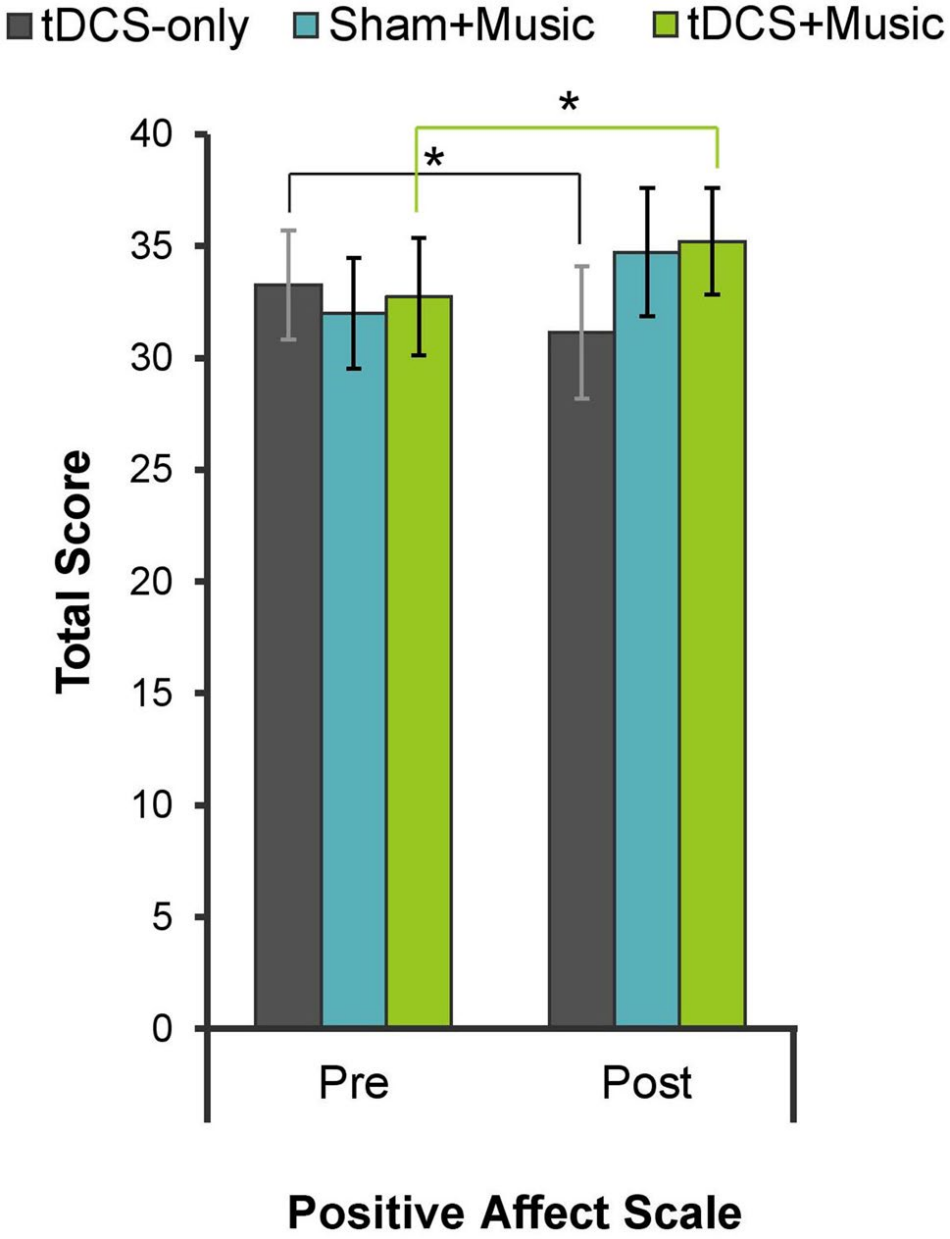
Change in PANAS ratings (post- minus pre-stimulation) by condition for positive and negative affect scales. Error bars represent standard error.

Positive affect ratings for the PANAS per condition are displayed in Fig. 1. The analysis revealed a significant Condition by Time interaction, *F*(2, 26) = 10.83, *p* < 0.001, *η*_*p*_^2^ = 0.454. Simple main effects analyses by Condition revealed, a significant decrease in positive affect ratings from pre- to post-stimulation in tDCS-only (*p* = 0.025), and a significant increase in positive affect ratings in tDCS + Music (*p* = 0.006). Positive affect ratings appeared to increase in Sham + Music from pre- to post-stimulation, but this was not significant (*p* = 0.106). Additional Sidak-corrected post-hoc pairwise comparisons of difference scores (post- minus pre-tDCS) revealed a greater change in positive affect ratings from pre- to post-tDCS in tDCS + Music (*p* = 0.013) and Sham + Music (*p* = 0.002) compared to tDCS-only. The change in positive affect ratings did not significantly differ between tDCS + Music and Sham + Music (*p* = 0.991).

### Digit span

**Figure 2:**
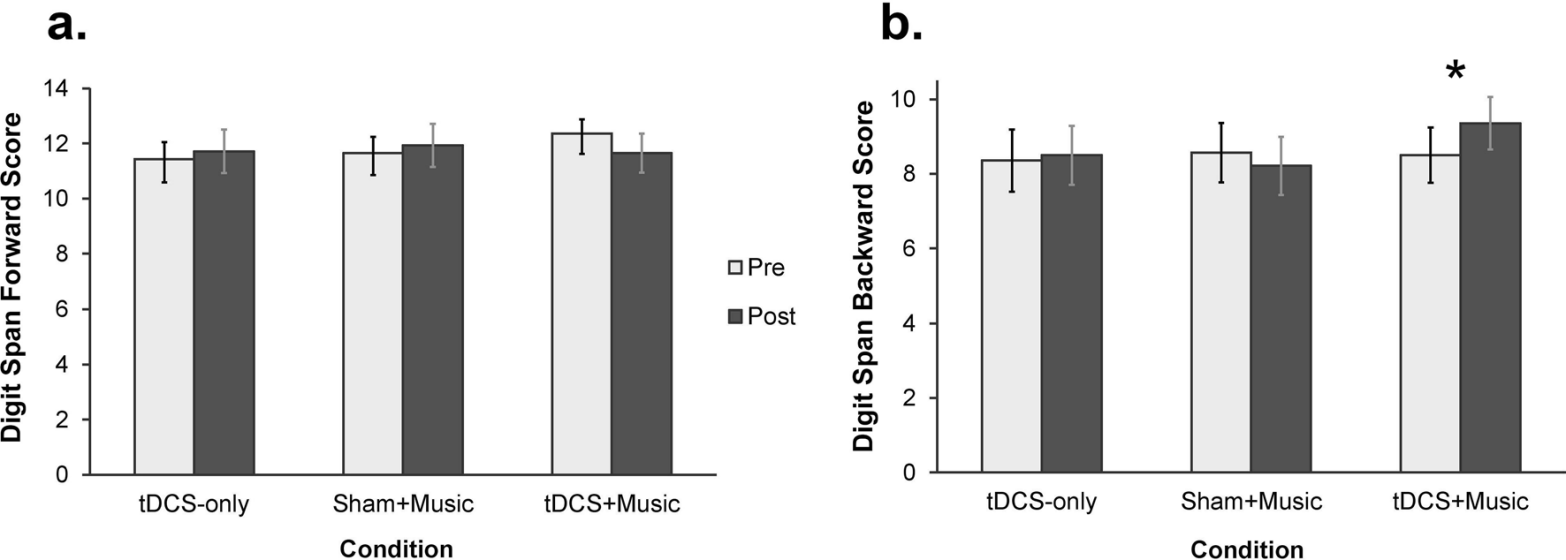
Digit span scores by condition for (**a**) forward and (**b**) backward subtests. Error bars represent standard error.

The measure of interest for the digit span was the change in working memory performance from pre- to post-stimulation for forward and backward scale (Fig. 2). For the digit span forward scale, there was no significant effect of Condition, *F*(2, 26) = 0.35, *p* = 0.771, *η*_*p*_^2^ = 0.026, Time, *F*(1, 13) = 0.02, *p* = 0.885, *η*_*p*_^2^ = 0.002, or Condition by Time interaction, *F*(2, 26) = 1.78, *p* = 0.188, *η*_*p*_^2^ = 0.121. For the digit span backward scale, the Condition by Time interaction was significant, *F*(2, 26) = 4.12, *p* = 0.028, *η*_*p*_^2^ = 0.241. As post-hoc power analyses using MorePower (version 6.0)^53^ showed a power of 68% for detecting a significant Condition by Time interaction given this effect size, we supplement pairwise comparisons with Bayes factors. Pairwise comparisons revealed a significant increase in backwards digit span score in the tDCS + Music condition (*p* = 0.012, B_10_ = 4.889), but no change in performance in the tDCS-only (*p* = 0.671, B_10_ = 0.293) or Sham + Music (*p* = 0.418, B_10_ = 0.365) conditions.

### WRT: behavioural measures

**Figure 3:**
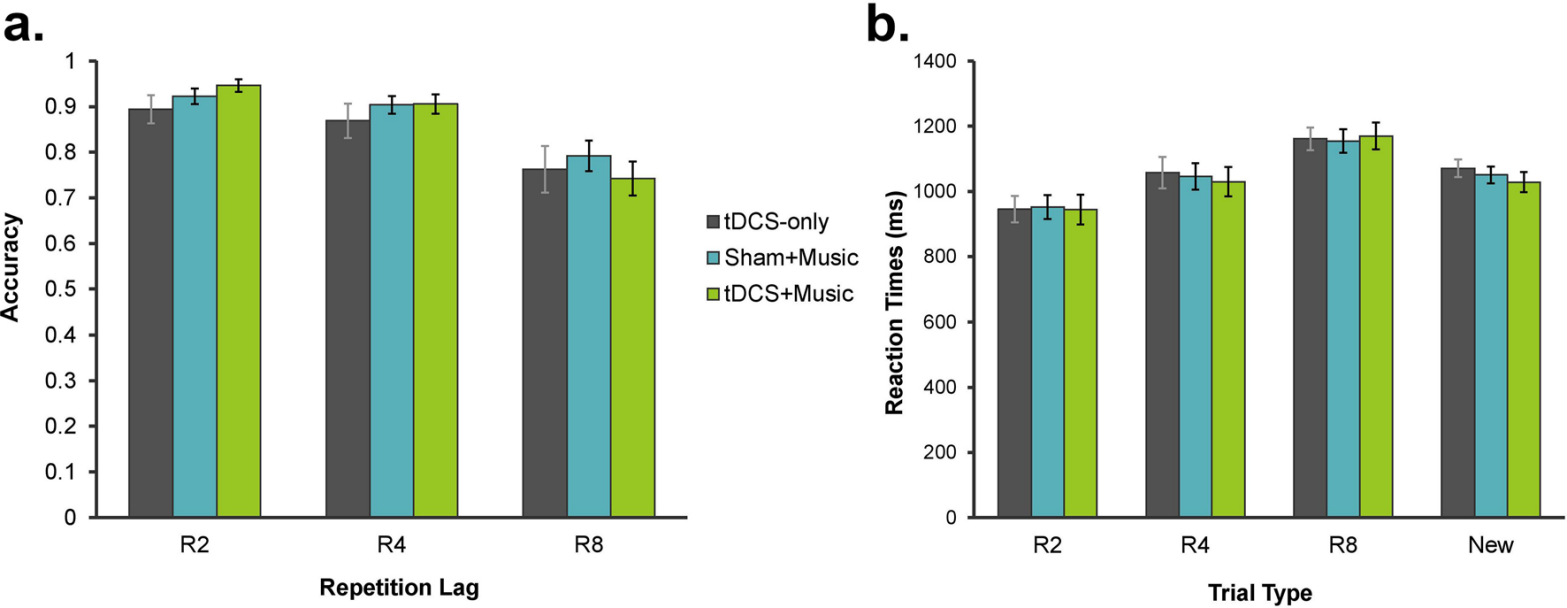
Performance on the auditory word recognition task across trial types in terms of (**a**) accuracy and (**b**) reaction times. Error bars represent standard error.

Accuracy and RT data for the auditory WRT are displayed in Fig. 3. The analysis for accuracy values showed a significant Condition by Trial Type interaction, *F*(4, 52) = 2.89, *p* = 0.031, *η*_*p*_^2^ = 0.182. Simple effects analyses by Trial Type revealed that this interaction was driven by a the effect of Condition that reached marginal significance in the R2 repetition lag, *F*(1.21, 15.69) = 3.54, *p* = 0.073, *η*_*p*_^2^ = 0.214. The effect of Condition was not significant in either the R4 (*F*(1.10, 14.29) = 0.90, *p* = 0.368, *η*_*p*_^2^ = 0.065) or R8 (*F*(2, 26) = 1.50, *p* = 0.243, *η*_*p*_^2^ = 0.103) repetition lags. The analysis also revealed a main effect of Trial Type, *F*(1.24, 16.09) = 21.44, *p* < 0.001, *η*_*p*_^2^ = 0.623. As expected, accuracy was lower for R8 than R4 (*p* = 0.001) and R2 (*p* = 0.002) repetition lags; accuracy was comparable across R2 and R4 repetition lags (*p* = 0.252).

Similarly, the analysis for RTs revealed a main effect of Trial Type, *F*(3, 39) = 13.67, p < 0.001, *η*_*p*_^2^ = 0.513. RTs were slower for old words in R8 than R4 (*p* < 0.001) and R2 (*p* = 0.001) repetition lags; RTs were also slower for old words in R4 than R2 repetition lags (*p* = 0.003). All pairwise comparisons between RTs for new words and RTs for old words did not reach significance (R2: *p* = 0.106; R4: *p* = 1.000; R8: *p* = 0.093). The main effect of Condition, *F*(2, 26) = 0.317, *p* = 0.731, *η*_*p*_^2^ = 0.024, and the Condition by Trial Type interaction, *F*(6, 78) = 1.14, *p* = 0.347, *η*_*p*_^2^ = 0.081, were not significant.

### WRT: ERP results

**Figure 4:**
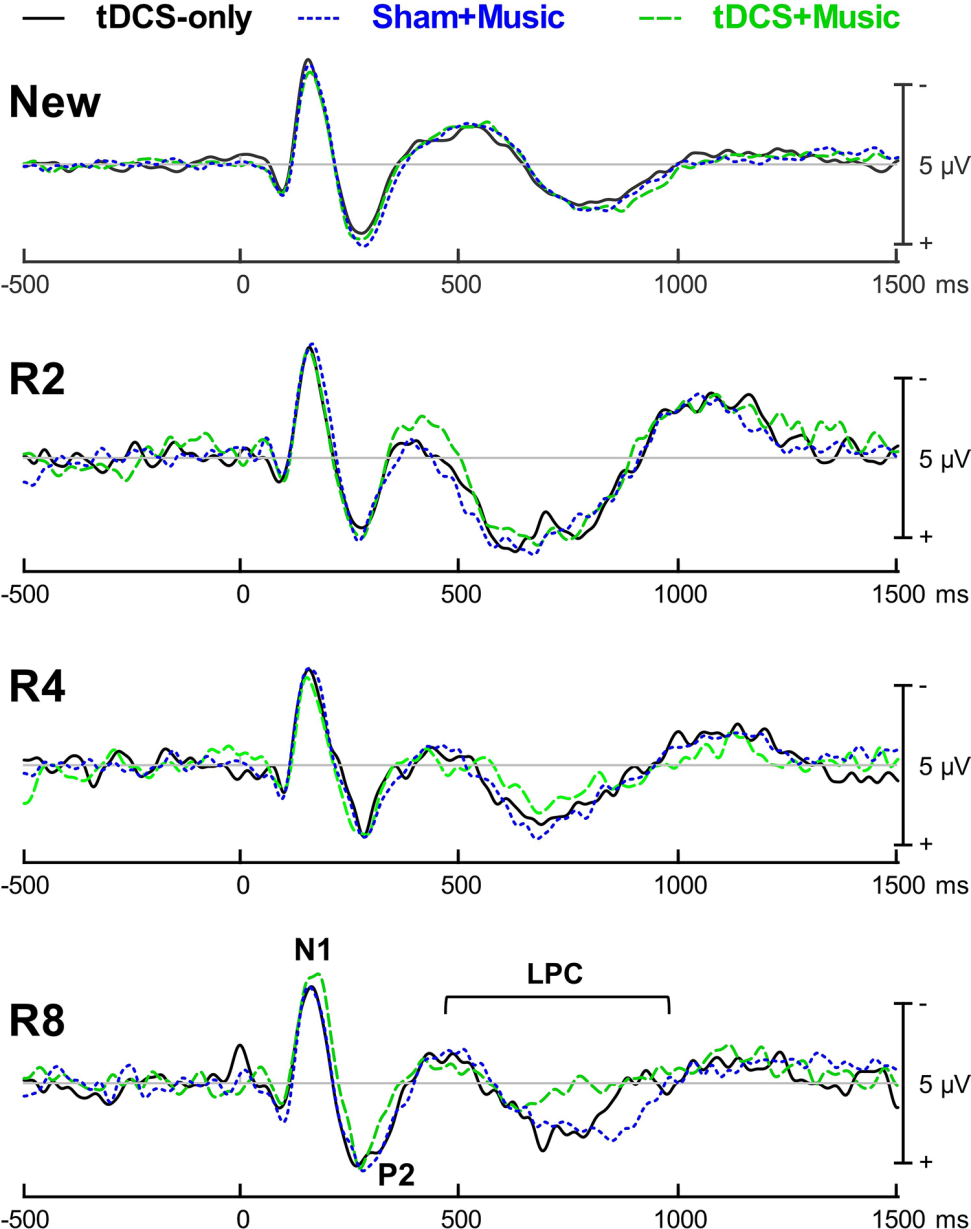
ERP waveforms by trial type for the auditory word recognition task averaged over the pre-defined centroparietal cluster (Cz, Pz, P3, and P4). Early auditory-evoked sensory potentials (i.e., P1, N2) are identified, as well as the Late Positive Complex (LPC). R2 = repetition lag with one intervening item; R4 = repetition lag with three intervening items; R8 = repetition lag with seven intervening items.

Figure 4 displays ERP waveforms by condition and repetition lag. The analysis for LPC peak latencies revealed a significant Condition by Trial Type interaction, *F*(6, 78) = 4.29, *p* = 0.001, *η*_*p*_^2^ = 0.248. Simple effects analyses by Trial Type revealed a significant main effect of Condition in the R8 repetition lag, *F*(2, 26) = 4.53, *p* = 0.021, *η*_*p*_^2^ = 0.258. Peak latencies for recognition of old words in the R8 repetition lag were significantly shorter in tDCS + Music than Sham + Music (*p* = 0.038); although latencies peaked earlier in tDCS + Music than that in tDCS-only, this comparison was not significant (*p* = 0.196). The effect of Condition was marginal for the R4 repetition lag, *F*(2, 26) = 3.09, *p* = 0.062, *η*_*p*_^2^ = 0.192, and non-significant for the R2 repetition lag *F*(2, 26) = 0.452, *p* = 0.641, *η*_*p*_^2^ = 0.034 or for new words *F*(2, 26) = 0.799, *p* = 0.460, *η*_*p*_^2^ = 0.058.

The analysis of LPC mean amplitudes revealed a significant Condition by Trial Type interaction, *F*(3.3, 43.0) = 5.71, *p* = 0.002, *η*_*p*_^2^ = 0.305. Post-hoc univariate ANOVAs of the old-new difference in amplitude revealed a significant main effect of Condition in the R8 repetition lag *F*(2, 26) = 6.11, *p* = 0.007, *η*_*p*_^2^ = 0.320. The old-new effect for R8 was significantly smaller in tDCS + Music than Sham + Music (*p* = 0.003), and appeared smaller in tDCS + Music than tDCS-only but was not significant (*p* = 0.155). The effect of Condition was marginally significant in the R4 repetition lag, *F*(2, 26) = 3.35, *p* = 0.051, *η*_*p*_^2^ = 0.205, and was not significant in the R2 repetition lag, *F*(2, 26) = 0.70, *p* = 0.505, *η*_*p*_^2^ = 0.051.

**Figure 5:**
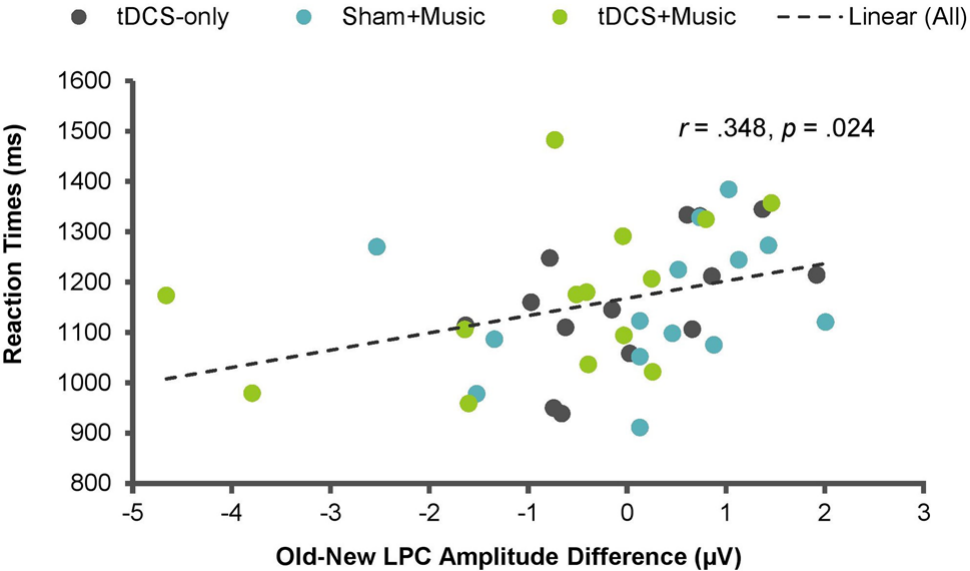
Scatter plot visualizing the correlation between the old-new Late Positive Complex (LPC) amplitude difference and RTs in the R8 repetition lag of the auditory word recognition task.

As LPC peak latencies and the old-new amplitude difference demonstrated a significant main effect of Condition, post-hoc two-tailed bivariate Pearson correlation analyses were run examining the brain-behaviour relationships averaged across Condition in the R8 repetition lag only. The analysis included two ERP measures (i.e., LPC peak latency and the old-new LPC amplitude difference) and two behavioural measures (accuracy or RTs). There was a significant positive correlation between the old-new amplitude difference and RTs (*r* = 0.348, *p* = 0.024) such that a greater old-new amplitude difference was associated with longer RTs (Fig. 5). The correlation between the old-new amplitude difference and accuracy did not reach significance (*r* = − 0.022, *p* = 0.891), and the correlations between peak latency and accuracy (*r* = 0.194, *p* = 0.219) and between peak latency and RTs (*r* = 0.080, *p* = 0.613) did not reach significance.

## Discussion

The present study explored whether combining anodal tDCS over the DLPFC with listening to autobiographically-salient music amplified changes in subsequent working memory and recognition memory performance in older adults compared to either music listening under sham stimulation or tDCS alone. Participants underwent three separate neurostimulation conditions (tDCS-only, Sham + Music, and tDCS + Music), in which working memory was assessed pre- and post-stimulation, and behavioural and electrophysiological measures of recognition memory were measured post-stimulation. In the tDCS + Music condition, older adults demonstrated an improvement in working memory between pre- and post-stimulation, but not in tDCS-only or Sham + Music conditions. In terms of subsequent recognition memory, older adults showed no significant differences in behavioural measures in any conditions; however, the LPC underlying the old-new effect in recognition memory in the R8 repetition lag differed between tDCS + Music and Sham + Music conditions, but not between tDCS + Music and tDCS-only.

For working memory, older adults in the present study demonstrated improvement in the backwards digit span with a small effect size in the combined tDCS + Music condition, and not in tDCS-only or Sham + Music conditions. To our knowledge, only one other study^34^ examined the effect of anodal tDCS on executive functioning by measuring response inhibition in younger adults pre- and post-tDCS in the presence of low-tempo and high-tempo background music, finding high-tempo background music to interact with tDCS on response inhibition. The present study extends this work to the domain of working memory and to healthy older adults. Despite several studies demonstrating tDCS-related improvements in working memory for younger adults^54–56^, this research with older adults remains limited. Some studies found improved verbal and visual working memory in older adults after anodal tDCS^28,57^ and when tDCS is paired with cognitive training^27^. One study^21^ also reported similar findings but only for older adults with high education. However, some studies find no change in working memory for older adults following tDCS with n-back tasks^30,58^, which are in accordance with the present study’s findings in the tDCS-only condition. Inconsistent findings are also present when examining tDCS effects in other cognitive domains, which may be attributed to differences in stimulation parameters, such as current intensity, duration of stimulation, and stimulation site^59^.

The literature examining the effects of music listening on working memory remains mixed as well. One study found improved performance on the forward digit span in older adults while listening to background classical music compared with white noise or silence^60^. However, it remains unclear whether this is attributed to increased arousal from music listening or some other attentional mechanism when cognitive performance is tested concurrently with background music. A more process pure conservative operationalization of the arousal-and-mood hypothesis describes mechanisms through which music may confer cognitive benefits following (rather than during) the listening of enjoyable music^1,2^. In examining subsequent working memory, studies have found no change in digit span performance in healthy older adults^61^ or older adults with amnestic mild cognitive impairment^62^ following listening to classical music. Similarly, one study^63^ found no change in working memory (with a reading span task) in older adults after listening to preferred music. Therefore, considering the mixed findings in either tDCS alone or music listening alone in improving working memory, the present study suggests that the pairing of tDCS with listening to autobiographically-salient music may amplify gains in working memory performance in older adults.

To our knowledge, this is the first study to examine recognition memory following tDCS and concurrent music listening. The WRT incorporated three repetition lags to measure recognition memory with increasing levels of task difficulty (i.e., increasing number of intervening items between word repetitions). In our accuracy and RT measures, we did not observe an effect of combining tDCS with listening to autobiographically-salient music. In our electrophysiological measures (i.e., LPC latency and amplitude), we observed earlier latencies for old words and a smaller old-new effect in amplitude for tDCS + Music than Sham + Music only for the R8 repetition lag. The smaller old-new effect in LPC amplitude for the R8 repetition lag was associated with faster RTs to correctly recognized words. Results of the correlations analyses suggest facilitated neural processing of word recognition in tDCS + Music relative to Sham + Music. Furthermore, earlier LPC latencies have been demonstrated in prior research to index faster recognition of familiar and recollected items^52,64^. These findings are consistent with previous meta-analyses demonstrating heightened processing speed in cognitive tasks after tDCS in healthy populations^65,66^. For old words, prior ERP research has also found smaller LPC amplitudes for old items with greater reoccurrence and greater semantic predictability^46,67^. Therefore, a smaller old-new effect in amplitude observed in the present study may index some neural trace of items held in working memory at the time of repetition, thereby reducing the need for recollection upon correct recognition of previously-heard words^46,67,68^. We emphasize that the present study, however, does not demonstrate behavioural differences in episodic memory when tDCS is paired with listening to autobiographically-salient music compared to the other experimental conditions.

Similar to the domain of working memory, the literature examining the effects of tDCS on older adults’ recognition memory (and other measures of episodic memory) has also been mixed. Prior research has shown improvements in verbal episodic memory in older adults following anodal stimulation of the DLPFC^23–26^, with some using other stimulation sites such as the ventrolateral prefrontal^69^ and the temporoparietal cortices^70^. However, several studies have also found null effects after stimulation over the DLPFC ^71^ or temporoparietal cortex^72^, and even impaired associative memory after stimulation of the left inferior frontal gyrus^73^. Nonetheless, a meta-analysis showed that tDCS strengthened episodic memory for older adults with moderate effect sizes^59^. The null accuracy and RT effects on the auditory WRT add to this mixed pattern of findings, and may reflect some limitations in our experimental design. For instance, the WRT was administered after a 15-min time delay during which participants were prepared for EEG recording, and the task lasted for another 15 min. The current intensity or the stimulation time may also have been too brief for a single stimulation session to yield observable behavioral effects in recognition memory. Furthermore, performance in the R2 and R4 repetition lags approached ceiling, which may have masked differences between neurostimulation conditions. Although some research has examined the effects of listening to autobiographically-salient music on subsequent episodic memory in younger adults^7,9^, this finding in healthy older adults is limited to one pilot study that found no change in verbal episodic memory after listening to non-preferred or preferred music^74^. Studies have examined episodic memory performance in older adults in the presence of concurrent background music during encoding or retrieval stages^6,75,76^, but it is unclear whether the observed changes are attributed to the arousal-and-mood hypothesis or some other attentional mechanism^1,2^. Our ERP findings suggest further exploration of combining tDCS with autobiographically-salient music in tasks with greater episodic memory demands (i.e., longer repetition lags).

The use of autobiographically-salient music may be optimal for exploring the arousal-and-mood hypothesis, as personally-meaningful music has been suggested to evoke autobiographical memories which thereby induce strong arousal and high ratings of affective positivity^10,11,77–82^. Indeed in the present study, ratings of positive affect increased after listening to autobiographically-salient music in the tDCS + Music condition (where we also observed improvement in working memory from pre- to post-tDCS). This is consistent with past research demonstrating increased arousal in older adults with physiological and subjective measures after listening to familiar individualized music^12,63,83,84^. Transcranial magnetic stimulation of the left DLPFC has also been shown to increase musical reward sensitivity through fronto-striatal pathways^85^. These pathways may also have been modulated through tDCS in the present study to further heighten arousal from music listening and thereby also heighten the effects of emotional arousal on cognitive performance. Furthermore, some individuals may experience mild side effects from neurostimulation and find the experience unenjoyable, which may hinder motivation and wash out tDCS-related benefits to cognitive performance. Indeed in the present study, ratings of positive affect decreased only in the tDCS-only condition. Pairing of tDCS with concurrent listening to personally-meaningful music may make the experience of tDCS more tolerable and enjoyable, and may distract users from tDCS-related side effects. Thus, the present study highlights the potential for autobiographically-salient music listening to heighten responsivity to tDCS in older adults, and may make the use of neurostimulation more appealing to new users.

The combination of neurostimulation and listening to autobiographically-salient music may have potential utility for clinical older adult populations who demonstrate a need for novel cognitive rehabilitation strategies, especially for individuals with Alzheimer’s disease (AD) and other dementias. Prior research has shown cognitive benefits in individuals with AD following listening to autobiographically-salient music, such as greater autobiographical recall, semantic fluency, and greater levels of social interaction and well-being^3,5,14,75,86,87^. Individuals with AD have also demonstrated cognitive benefits with anodal tDCS, particularly in the domains of working memory and episodic memory^17,29,88–90^ with greater effect sizes than healthy older adults^17^. Future research may explore the clinical utility of combining non-invasive neurostimulation with concurrent listening to autobiographically-salient music as a potential framework for cognitive rehabilitation for working memory in older adults with and without cognitive impairment.

Some limitations of the present study include the experimenters’ lack of blinding to neurostimulation condition during administration of cognitive tasks. Furthermore, as we only measured recognition memory post-stimulation, the study is limited in comparing the independent contributions to recognition memory performance from tDCS alone or autobiographically-salient music alone compared with baseline performance. This study also employed a small sample size, although this size was similar to those of past studies with tDCS and music listening^34,35^. The generalizability of these findings may also be limited by the high level of education in our sample and the greater female to male ratio—with the latter being of note as responsivity to tDCS has been found to be greater for females than males^66^. The additional use of electrodermal measures would better quantify arousal levels during music listening^34^. Given these limitations, we recommend careful interpretation of the present results, and for future research to examine the effects of tDCS with larger sample sizes and how demographic variables such as sex and education moderate these effects. Moreover, future studies should investigate the durability of such effects to better address the clinical relevance of these techniques for older adults. These future studies may opt to use a within-subjects design such as the present study, as this design would account for individual differences such as musical training or the vocal/instrumental nature of personalized playlists between music listening conditions.

## Concluding remarks

The present study suggests that combining tDCS with listening to autobiographically-salient music may amplify gains in working memory relative to either tDCS alone or music listening alone. Although no evidence for behavioural benefits in recognition memory was found, the combination of tDCS with music listening may modulate neural processing of recognition memory compared to sham stimulation when episodic memory demands are high. This study suggests that listening to music, particularly to songs that evoke autobiographical memories, may heighten responsivity to the effects of tDCS for healthy older adults, and may maximize the tolerability of an individual’s experience with tDCS. Findings from this present study may inform how to maximize the responsivity of neurostimulationsing personalized music for older adult users of tDCS by using personalized music.

## Data Availability

This data can be made available upon reasonable request from the corresponding author and in accordance with applicable laws.
